# The utility of coagulation activity for prediction of risk of mortality and cardiovascular events in guideline-treated myocardial infarction patients

**DOI:** 10.1080/03009734.2017.1407849

**Published:** 2018-01-04

**Authors:** Christina Christersson, Bertil Lindahl, Lars Berglund, Agneta Siegbahn, Jonas Oldgren

**Affiliations:** a Department of Medical Sciences, Cardiology, Uppsala University, Uppsala, Sweden; b Uppsala Clinical Research Center, Uppsala University, Uppsala, Sweden; c Department of Medical Sciences, Clinical Chemistry, Uppsala University, Uppsala, Sweden

**Keywords:** D-dimer, heart failure, myocardial infarction, thrombin, thromboembolism

## Abstract

**Background:**

Despite improved treatment of myocardial infarction (MI), real-world patients still suffer substantial risk for subsequent cardiovascular events. Little is known about coagulation activity shortly after MI and whether coagulation activity markers may identify patients at increased risk despite contemporary treatment.

**Objective:**

To evaluate D-dimer concentration and thrombin generation potential shortly after discharge after MI and evaluate if these markers could predict the risk of future cardiovascular and bleeding events.

**Methods:**

Unselected MI patients (*n* = 421) were included in the observational REBUS study (NCT01102933) and followed for two years. D-dimer concentrations, thrombin peak, and endogenous thrombin potential (ETP) were analyzed at inclusion (3–5 days after MI) and at early follow-up (after 2–3 weeks).

**Results:**

Seventy-five patients (17.8%) experienced the composite endpoint (all-cause death, MI, congestive heart failure, or all-cause stroke), and 31 patients (7.4%) experienced a clinically relevant bleeding event. D-dimer concentrations at early follow-up were associated with the composite endpoint (HR [per SD increase] 1.51 [95% CI 1.22–1.87]) and with clinically relevant bleeding (HR [per SD increase] 1.80 [95% CI 1.32–2.44]). Thrombin generation potential was not significantly associated with either the composite endpoint or with clinically relevant bleeding. Higher thrombin peak and ETP at early follow-up were both inversely associated with stroke (HR [per SD increase] 0.50 [95% CI 0.30–0.81] and 0.43 [95% CI 0.22–0.83], respectively).

**Conclusion:**

In unselected MI patients treated according to contemporary guidelines, D-dimer measurements may identify patients at increased risk of new cardiovascular and bleeding events. The inverse association of thrombin generation potential and risk of stroke has to be further investigated.

## Introduction

Early outcome of acute myocardial infarction (MI) has been improved during the last decades. More effective treatment strategies have been developed, such as parenteral anticoagulation therapy, early revascularization procedures, and secondary prevention with dual antiplatelets, angiotensin-converting enzyme inhibitors, and statins. However, despite receiving treatment according to guidelines, real-world patients still suffer a substantial risk for new cardiovascular events during follow-up (1).

In studies performed before the era of dual antiplatelet therapy and early revascularization, patients with acute coronary syndromes (ACS) were shown to have increased coagulation activity after the acute event, and the correspondingly high D-dimer concentrations were associated with increased risk of recurrent ACS (2). Early reduction of D-dimer concentrations was associated with decreased risk of new cardiovascular events (3). However, long-term dual compared to single antiplatelet treatment after MI does not further reduce D-dimer concentrations (4). In addition, long-term oral anticoagulant treatment has been shown to reduce coagulation activity in ACS patients and decrease the risk of cardiovascular events but to increase the risk of bleeding events (5,6).

The above-mentioned studies on coagulation activity after ACS, including MI, were mainly performed in highly selected patient groups participating in randomized clinical trials evaluating new medical treatment. The results of these studies may therefore not be applicable to the unselected real-world MI patient group (7). MI patients not participating in clinical trials are often older and have more comorbidities, factors which have been associated with increased coagulation activity (8).

We therefore evaluated coagulation activity early after MI in a cohort of unselected patients and investigated whether coagulation activity, measured as D-dimer concentrations and thrombin generation potential, could predict the risk of future cardiovascular and bleeding events.

## Material and methods

### Patient population

**Figure 1.**
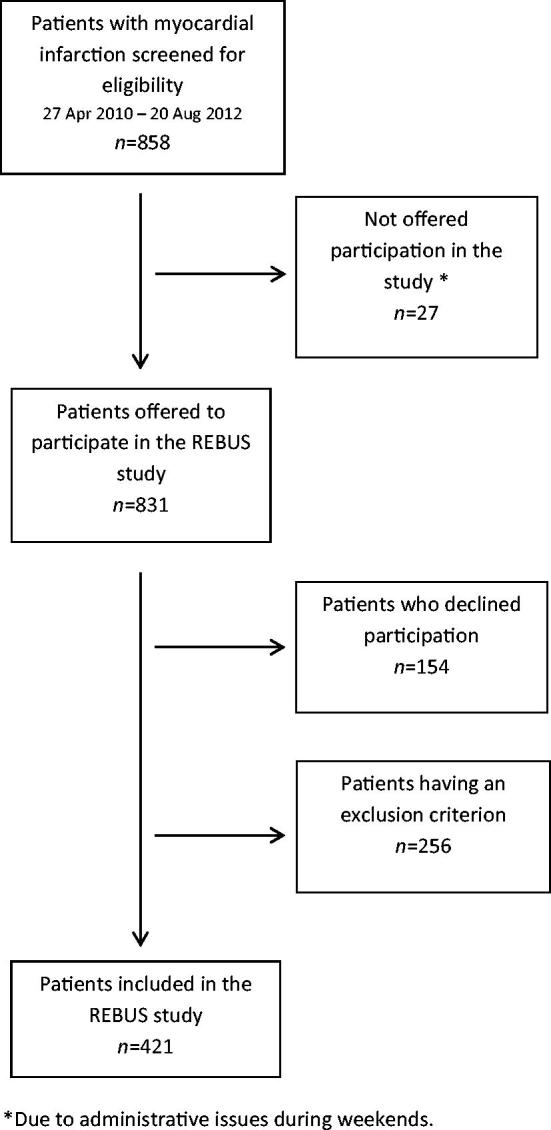
A flow chart of the patients included in the REBUS study.

The REBUS (The RElevance of Biomarkers for future risk of thromboembolic events in UnSelected post-myocardial infarction patients) study was a prospective observational study of patients with recent ACS (NCT01102933, ClinicalTrials.gov). Patients with myocardial infarction (MI), both non-ST-elevation (NSTEMI) and ST-elevation (STEMI) MI, admitted to the acute coronary care unit at the Department of Cardiology, Uppsala University Hospital, during 2010–2012 were included. The inclusion criterion was MI diagnosed as a dynamic raise in troponin I with at least one value above the decision limit for MI together with at least one of the following criteria: (1) symptoms suggestive of MI, and (2) development of a significant Q wave. Exclusion criteria were death ≤5 days after MI, living outside the catchment area of Uppsala University Hospital, or lack of suitability for participation in the trial for any reason, including inability to attend the scheduled study visits for evaluation procedures as judged by the investigator. Consecutive enrollment of patients was strongly encouraged, although sometimes limited due to practical constraints at the coronary care unit (Figure 1). Patients were included 3–5 days after the index MI, before discharge from the hospital, and followed for two years. An early follow-up visit was performed at 2–3 weeks after inclusion in the study, and subsequent follow-up visits were at 3, 12, and 24 months after the index event. Patients were treated according to international and national guidelines, at the discretion of the responsible physicians. The study was approved by the local ethics committee and followed the regulations of the Helsinki declaration.

### Clinical endpoints

The composite of cardiovascular endpoints consisted of all-cause death, new myocardial infarction, all-cause stroke, and congestive heart failure. Deaths were further subclassified as cardiovascular or non-cardiovascular. Deaths from cardiovascular causes included cardiac and cerebrovascular deaths as well as other vascular abnormalities. Deaths from unknown/uncertain causes were categorized as cardiovascular deaths. New myocardial infarctions were defined in the same way as the index MI. Stroke was diagnosed as abrupt onset of focal neurological deficit persisting more than 24 hours and assessed by computed tomography or magnetic resonance imaging scan and included both ischemic and hemorrhagic strokes. Strokes were further subclassified as ischemic or hemorrhagic stroke. Congestive heart failure (CHF) was defined as hospitalization due to symptoms suggestive of heart failure, which had to be verified with objective findings by lung X-ray, echocardiography, or increased levels of NT-proBNP. Thus, the CHF endpoint included both new-onset CHF and worsening of CHF in patients with a medical history of CHF. A clinically relevant bleeding event, excluding hemorrhagic stroke, was defined as a bleeding event leading to hospital admission for clinical evaluation and medical or surgical treatment as indicated, in line with the International Society of Thrombosis and Haemostasis (ISTH) definition of clinically relevant non-major bleeding events. At every study visit, the patients were asked for symptoms or signs suggestive of any of the outcome events since the previous study visit, and the medical records were evaluated to identify outcome events. Endpoints were not formally adjudicated; however, after the last study visit, a study physician evaluated all potential outcomes for each patient. After study completion, clinical monitors from Uppsala Clinical Research Center scrutinized the medical records of all patients for identification of potentially missed outcomes.

### Plasma analysis of D-dimer concentrations and thrombin generation potential

Blood was collected in citrate tubes by direct puncture with no stasis at inclusion in the study 3–5 days after index MI and at early follow-up 2–3 weeks after the index event. After centrifugation, platelet-poor plasma was stored at –80 °C until analysis.

D-dimer concentrations were assessed using an enzyme immunoassay (Asserachrome, Stago, France). The reference interval was <500 µg/L. The coefficient of variances was 11%. D-dimer results were available in 98% and 95% of the patients at inclusion and at early follow-up, respectively.

Thrombin generation potential was assessed by the Calibrated Automated Thrombogram (Thrombinoscope) measured in a 96-well plate fluorometer (Fluoroskan Ascent^®^, ThermoScientific, Waltham, MA, USA) with previously described modifications (9). Eighty microliter plasma were mixed with 20 µL of Hepes-buffered saline with bovine serum albumin (BSA) (pH 7.35, 20 nM Hepes, 140 mM NaCl, 5 mg/mL BSA). Samples spiked with 20 µL Thrombin Calibrator (Thrombinoscope) were run in parallel with each cycle of test samples. Samples were run in triplicate. The fluorometric measurements were performed after automated addition of 20 µL FluCa-kit (417 µM fluorescent substrate Z-Gly-Gly-Arg-AMC and 16.7 nM CaCl_2_, final concentrations). No exogenous tissue factor or phospholipids were added to the assay. The thrombin generation process was monitored for 120 minutes. The peak of thrombin generation and the endogenous thrombin potential (ETP) were calculated. The coefficients of variances were 15% and 10% for thrombin peak and ETP, respectively. Analyses without a significant curve for thrombin generation were discarded; thereby thrombin generation results were available from 77% and 81% of the study cohort at inclusion and early follow-up, respectively. All analyses were performed at the Uppsala Clinical Research Center (UCR) laboratory, Uppsala, Sweden.

### Statistical methods

The sample size was based on results from ESTEEM, a randomized clinical trial of patients with MI (3). In ESTEEM, 60% of patients had early decreased D-dimer values, which was associated with a 9% incidence of composite cardiovascular event, i.e. death, MI, severe recurrent ischemia, and stroke. In patients with unchanged or increased D-dimer concentration (40% of the study population), the incidence of cardiovascular events was 16%. We estimated the total incidence of the composite of cardiovascular endpoints in the REBUS population to be 20% due to higher event rates in an unselected population. We further estimated the incidence of events to be 15% in patients with decreased D-dimer values and to be 27% in patients with unchanged or increased D-dimer concentration after MI.

Based on Fisher’s exact test, with 5% significance level and a power of 80%, 403 patients were required. To compensate for premature withdrawals, 421 patients were included in the REBUS study.

Continuous variables were described by medians and interquartile ranges or by means and standard deviations. Categorical variables were described by frequencies and percentages. Continuous variables were compared between groups with the Mann–Whitney test and with 95% confidence intervals for median group differences. Continuous variables were compared between visits with Wilcoxon’s matched-pairs signed rank test.

For continuous variables, the Shapiro–Wilk test statistic W was calculated where the region W ≥ 0.95 implied use of the original scale (ETP) and W < 0.95 indicated use of the logarithmic scale (D-dimer and thrombin peak) of the variable in the Cox proportional hazards regression models.

The endpoints were the composite of all-cause death, new MI, CHF, and all-cause stroke; the individual components of the composite; and clinically relevant bleeding event. Time to event was measured from the date of inclusion after the index MI for associations with biomarkers at inclusion, and from date for early follow-up for associations with biomarkers at early follow-up, and during the follow-up time up to a maximum of two years. For the composite endpoint time to first event of any of the individual components was calculated. Relations between biomarkers and endpoints were investigated with Cox proportional hazards regression models and presented as hazard ratios with 95% confidence intervals of one standard deviation increase of the respective biomarker and *P* values. Proportional hazards assumptions of Cox regression models were confirmed with Schoenfeld residual test.

Cox regression models were estimated, for each biomarker and endpoint, as univariate models and with adjustments for established risk factors measured at baseline (age, sex, hypertension, type 2 diabetes, atrial fibrillation, previous congestive heart failure, and MI type [NSTEMI/STEMI]) (model 1), and antithrombotic treatments at inclusion and, for biomarkers measured at the early follow-up visit, antithrombotic treatments at the early follow-up visit were also used as covariates (model 2). Model 3 included model 1 and model 2.

All statistical tests and confidence intervals were two-sided (where applicable). Results with *P* values <0.05 were considered statistically significant without adjustments for multiplicity. The statistical analyses were performed with the statistical program package SAS version 9.4 (SAS Institute Inc., Cary, NC, USA).

## Results

### Baseline characteristics

The REBUS study included 421 patients during 2010–2012. Median (interquartile range) from index MI to inclusion was 2.0 (2.0–3.0) days. Patient baseline characteristics are described in Table I. Age and sex distribution in the study population was comparable to the national SWEDEHEART registry (10). The index MI was NSTEMI in 227 patients (53.9%) and STEMI in 194 patients (46.1%). A coronary angiogram was performed in 96.2% of patients, 81.7% of patients underwent a percutaneous coronary intervention (PCI) during hospital stay, and 2.2% were scheduled for coronary artery bypass grafting (CABG). Echocardiography was performed in 95.2% of patients, of whom 26.9% had moderate to severe reduction of the left ventricular ejection fraction (LVEF).

**Table 1.** Baseline characteristics of the REBUS population and pharmaceutical treatment at hospital discharge. Results describe number of patients (proportion) unless stated otherwise.

	REBUS population (*n* = 421)	NSTEMI (*n* = 227)	STEMI (*n* = 194)
Age, mean (SD)	67.0 (10.3)	67.6 (10.6)	66.3 (10.0)
<60 years	103 (24.5)	51 (22.5)	52 (26.8)
60–69 years	164 (39.0)	85 (37.4)	79 (40.7)
70–79 years	105 (24.8)	61 (26.9)	44 (22.7)
≥80 years	49 (11.6)	30 (13.2)	19 (9.8)
Sex			
Female	94 (22.3)	57 (25.1)	37 (19.1)
Male	327 (77.7)	170 (74.9)	157 (80.9)
Current smokers	108 (25.7)	40 (17.6)	68 (35.1)
BMI (kg/m^2^), mean (SD)	27.3 (4.2)	27.7 (4.4)	26.9 (3.9)
Waist circumference (cm), mean (SD)	101.8 (11.4)	102.5 (12.3)	101.0 (10.2)
Diabetes^a^	67 (15.9)	40 (17.6)	27 (13.9)
Hypertension	267 (63.4)	161 (70.9)	106 (54.6)
Previous myocardial infarction	86 (20.4)	62 (27.3)	24 (12.4)
Previous stroke	21 (5.0)	12 (5.3)	9 (4.6)
History of peripheral arterial disease	12 (2.9)	7 (3.1)	5 (2.6)
Atrial fibrillation	37 (8.8)	25 (11.0)	12 (6.2)
History of congestive heart failure	31 (7.4)	20 (8.8)	11 (5.7)
Pharmaceutical treatment at hospital discharge after index event:		
Aspirin	413 (98.1)	220 (96.9)	193 (99.5)
ADP receptor-blocking agent	406 (96.1)	213 (93.8)	193 (99.5)
Clopidogrel	314 (74.6)	166 (73.1)	148 (76.3)
Ticagrelor	88 (20.9)	45 (19.8)	43 (22.2)
Prasugrel	7 (1.7)	3 (1.3)	4 (2.1)
Oral anticoagulant treatment^b^	27 (6.4)	16 (7.0)	11 (5.7)
Statins	396 (94.1)	211 (93.0)	185 (95.4)
ACEi/ARB	336 (79.8)	171 (75.3)	165 (85.1)
Beta receptor-blocking agent	391 (92.9)	207 (91.2)	184 (94.8)
Calcium channel-blocking agent	53 (12.6)	44 (19.4)	9 (4.6)
Long-acting nitrate	44 (10.5)	35 (15.4)	9 (4.6)
Anti-diabetic drugs			
Oral	43 (10.2)	26 (11.5)	17 (8.8)
Insulin	34 (8.1)	26 (8.8)	14 (7.2)

a Diabetes, including all subtypes.

b Oral anticoagulant treatment; only warfarin was used during the period of this trial.

ACEi: angiotensin-converting enzyme inhibitor; ARB: angiotensin II receptor-blocking agent.

### Pharmaceutical treatment at discharge and at early follow-up

At discharge, >95% of patients had been prescribed dual antiplatelet treatment, 6.4% oral anticoagulant treatment (OAC), >90% statins, and almost 80% an ACE inhibitor (ACEi) or angiotensin II receptor-blocking agent (ARB) (Table 1). At the early follow-up visit, 98% were still on treatment with aspirin, 96% on an ADP receptor-blocking agent, 6.4% on an OAC, 94% on a statin, and 85% of the patients on ACEi/ARB.

### Clinical endpoints

The composite endpoint (all-cause death, new MI, all-cause stroke, or CHF) occurred in 75 patients (17.8%) during the two-year follow-up period. Fourteen patients (3.3%) died, of whom six from cardiovascular causes. In the total patient cohort, 36 patients (8.6%) suffered a new MI. Eleven patients (2.6%) had a stroke, of whom six were subclassified as ischemic and five as hemorrhagic. Hospitalization for CHF occurred in 31 patients (7.4%), and clinically relevant bleeding events occurred in 31 patients (7.4%).

### D-dimer concentrations at inclusion and at early follow-up after myocardial infarction

Median (interquartile range) of D-dimer concentrations were 677 µg/L (449–1137 µg/L) at inclusion and 615 µg/L (424–1150 µg/L) at early follow-up (*P* = 0.65) (Table 2). Older age and previous stroke were associated with higher D-dimer concentrations at early follow-up, while statin treatment was associated with lower D-dimer concentrations (Table 3).

**Table 2.** D-dimer concentrations, thrombin peak concentrations, and ETP given as median (interquartile range).

	Inclusion 3–5 days after MI	Early follow-up 2–3 weeks after discharge	Absolute change from inclusion to early follow-up
D-dimer (µg/L)	677 (449; 1137) *n* = 412	615 (424; 1150) *n* = 399	−6.5 (−174; 172) *P* = 0.65
Thrombin peak (nM)	60 (30; 101) *n* = 323	57 (31; 90) *n* = 343	−7.0 (−38; 28) *P* = 0.07
ETP (AUC)	1098 (672; 1388) *n* = 323	1047 (668; 1308) *n* = 343	−85 (−385; 196) *P* = 0.011

**Table 3.** Biomarkers at early follow-up (2–3 weeks after discharge) in relation to baseline characteristics and pharmaceutical treatment at hospital discharge after index event.

	D-dimer (µg/L)		Thrombin peak (nM)		ETP (AUC)	
	Median diff. (95% CI)^a^	*P* value^b^	Median diff. (95% CI)^a^	*P* value^b^	Median diff. (95% CI)^a^	*P* value^b^
Clinical characteristics:						
Age: <67 years/≥67 years	−372 (−507; −238)	<0.0001	−6 (−17; 6)	0.34	−48 (−181; 85)	0.48
Sex: Female/Male	60 (−100; 219)	0.46	2 (−10; 15)	0.70	−12 (−172; 148)	0.88
Smoking: Yes/No	−17 (−141; 107)	0.79	4 (0; 7)	0.05	27 (−109; 164)	0.70
Index MI: NSTEMI/STEMI	−61 (−180; 58)	0.32	6 (−7; 19)	0.38	31 (−100; 162)	0.64
Diabetes mellitus: Yes/No	−14 (−199; 171)	0.88	13 (−7; 33)	0.20	16 (−146; 179)	0.84
Hypertension: Yes/No	−14 (−146; 119)	0.84	−4 (−16; 8)	0.53	−66 (−190; 57)	0.29
Previous MI: Yes/No	59 (−120; 238)	0.52	14 (−2; 31)	0.08	35 (−87; 157)	0.57
Previous stroke: Yes/No	468 (26; 946)	0.0385	−16 (−45; 13)	0.28	−174 (−658; 312)	0.48
History of peripheral artery disease: Yes/No	432 (−550; 1415)	0.39	−5 (−84; 74)	0.90	123 (−552; 798)	0.72
Atrial fibrillation: Yes/No	169 (−142; 480)	0.29	−17 (−53; 19)	0.35	−168 (−545; 209)	0.38
Congestive heart failure: Yes/No	474 (−389; 1337)	0.28	−15 (−38; 8)	0.20	88 (−242; 419)	0.60
Pharmaceutical treatment at hospital discharge:						
OAC: Yes/No	−163 (−525; 199)	0.38	−41 (−71; −11)	0.0082	710 (−1266; −155)	0.0121
Statins: Yes/No	−450 (−837; −62)	0.0231	0 (−9; 10)	0.96	63 (−197; 323)	0.63
ACEi/ARB: Yes/No	−12 (−154; 129)	0.87	−10 (−21; 1)	0.08	−126 (−237; −16)	0.0251

a The median difference (95% CI) of the biomarkers at early follow-up in the group with and without the clinical characteristics or treatment.

b *P* value from Mann−Whitney’s test.

ACEi: angiotensin-converting enzyme inhibitor; ARB: angiotensin II-blocking agent; MI: myocardial infarction; NSTEMI: non-ST-elevation myocardial infarction; OAC: oral anticoagulant treatment; STEMI: ST-elevation myocardial infarction.

### D-dimer concentrations and risk of future cardiovascular and bleeding events

**Figure 2.**
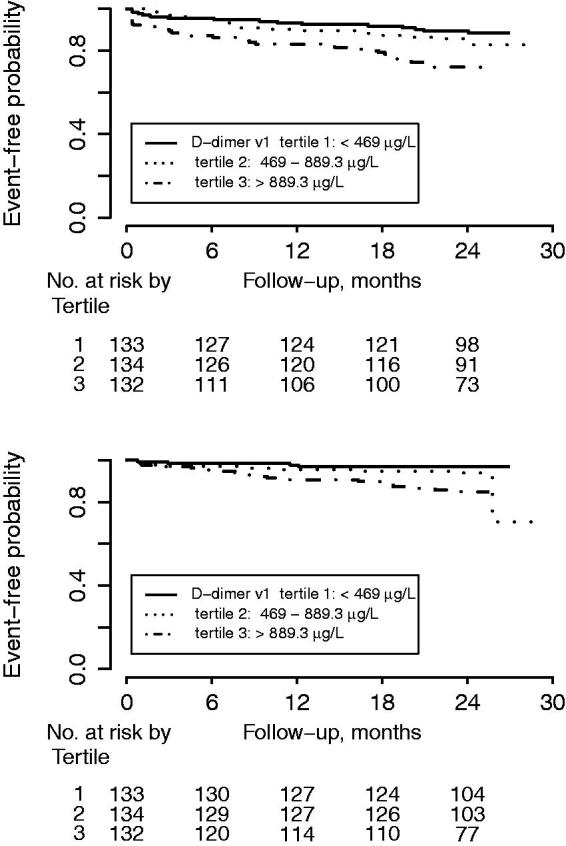
Kaplan–Meier estimate for (upper panel) the composite endpoint of all-cause mortality, new myocardial infarction, congestive heart failure, and all-cause stroke; and (lower panel) clinically relevant bleeding events by D-dimer concentration tertiles.

**Figure 3.**
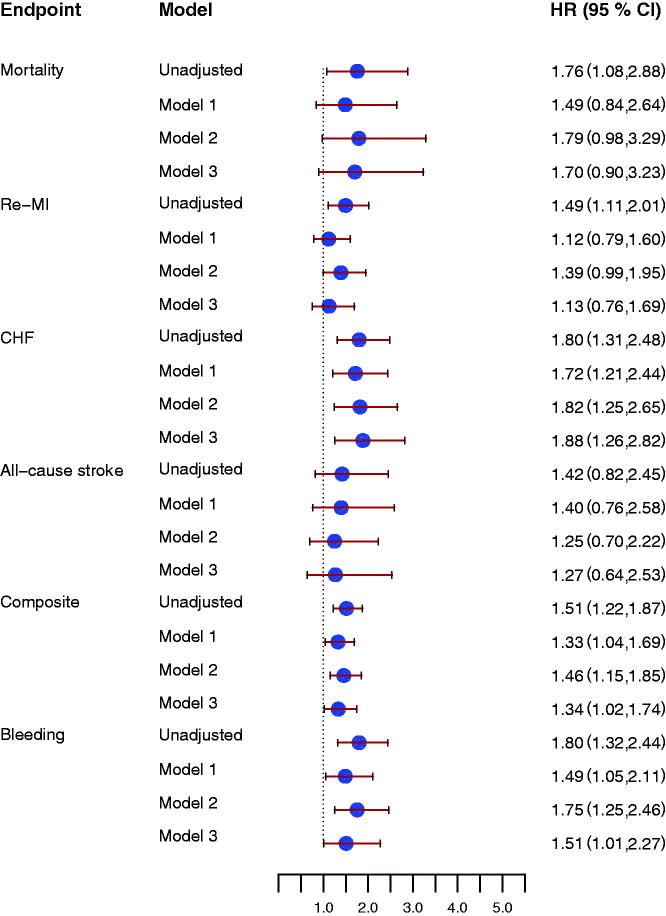
Forrest plot for the effect of D-dimer concentrations at early follow-up for prediction of all-cause mortality, new myocardial infarction (re-MI), congestive heart failure (CHF), all-cause stroke, the composite endpoint, and clinically relevant bleeding event (Bleeding), unadjusted and after adjustment. Model 1 included adjustment for age, sex, hypertension, diabetes, atrial fibrillation, previous congestive heart failure, NSTEMI/STEMI as index event. Model 2 included antithrombotic treatment: aspirin, ADP receptor-blocking agent, and oral anticoagulant treatment at inclusion and at the early follow-up visit. Model 3 included model 1 and model 2. All hazard ratios reflect the effect of a one standard deviation increase.

D-dimer concentrations at inclusion were not associated with the composite endpoint (HR 1.22 [95% CI 0.99–1.51], *P* = 0.06, for one SD increase). D-dimer concentrations at early follow-up were significantly associated with the composite endpoint (HR 1.51 [95% CI 1.22–1.87], *P* = 0.0001) (Figure 2, upper panel). In unadjusted analyses, D-dimer concentrations at early follow-up were associated with all-cause mortality, new MI, and CHF, but not with all-cause stroke (Figure 3). Associations were attenuated after adjustment for baseline characteristics, but D-dimer concentrations were still associated with the composite endpoint and with CHF. Similar results were found after adding antithrombotic treatment to the statistical model (Figure 3). Changes in D-dimer concentrations from inclusion to early follow-up were not associated with either the composite endpoint or with the individual cardiovascular endpoints.

In unadjusted analyses, D-dimer concentrations at inclusion, at early follow-up, as well as the changes in D-dimer concentrations between inclusion and early follow-up were all associated with increased risk of clinically relevant bleeding. After adjustment for baseline characteristics only early follow-up D-dimer concentrations were still associated with increased bleeding risk (Figure 2, lower panel, and Figure 3).

### Thrombin generation potential at inclusion and at early follow-up after myocardial infarction

Median thrombin peak (interquartile range) was 60 (30–101) nM at inclusion and 57 (31–90) nM at early follow-up (*P* = 0.07) (Table 2). The median ETP (interquartile range) was 1098 (672–1388) at inclusion and 1047 (668–1308) at early follow-up (*P* = 0.011) (Table 2). Neither the thrombin peak at early follow-up nor ETP was associated with baseline characteristics. In contrast, treatment with OAC and ACEi/ARB was related to lower levels of thrombin peak and ETP at early follow-up (Table 3).

### Thrombin generation potential and risk of future cardiovascular and bleeding events

**Figure 4.**
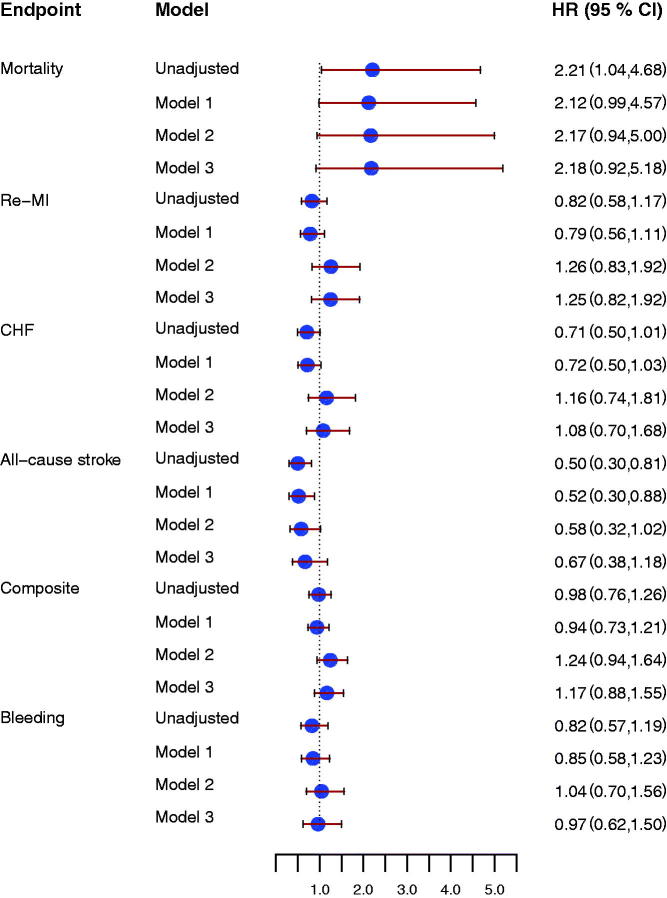
Forrest plot for the effect of thrombin peak at early follow-up for prediction of all-cause mortality, new myocardial infarction (re-MI), congestive heart failure (CHF), all-cause stroke, the composite endpoint, and clinically relevant bleeding event (Bleeding), unadjusted and after adjustment. Model 1 included adjustment for age, sex, hypertension, diabetes, atrial fibrillation, previous congestive heart failure, NSTEMI/STEMI as index event. Model 2 included antithrombotic treatment: aspirin, ADP-receptor blocking agent, and oral anticoagulant treatment at inclusion and at the early follow up visit. Model 3 included model 1 and model 2. All hazard ratios reflect the effect of a one standard deviation increase.

**Figure 5.**
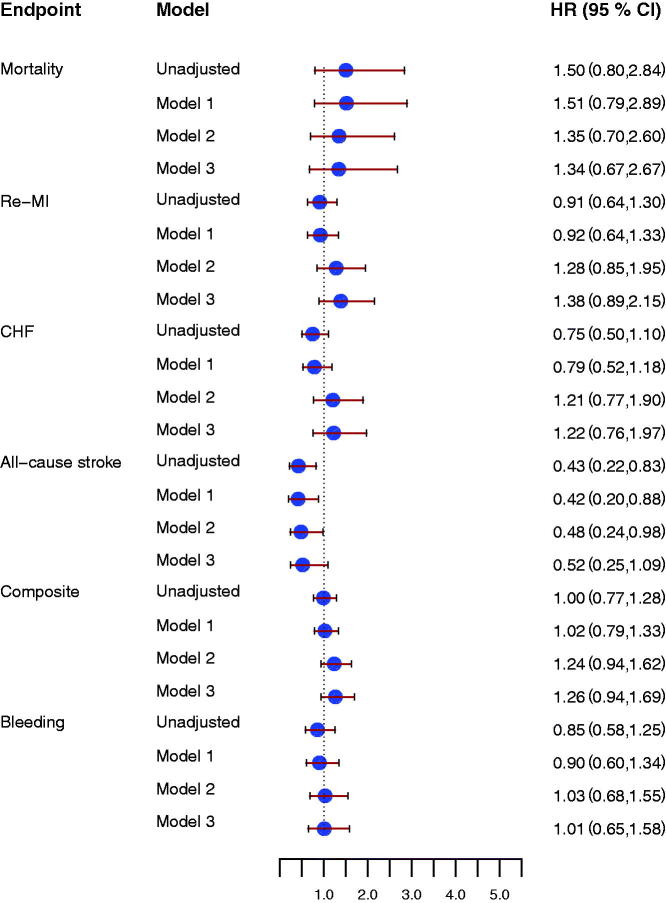
Forrest plot for the effect of endogenous thrombin potential (ETP) at early follow-up for prediction of all-cause mortality, new myocardial infarction (re-MI), congestive heart failure (CHF), all-cause stroke, the composite endpoint and clinically relevant bleeding event (Bleeding), unadjusted and after adjustment. Model 1 included adjustment for age, sex, hypertension, diabetes, atrial fibrillation, previous congestive heart failure, NSTEMI/STEMI as index event. Model 2 included antithrombotic treatment: aspirin, ADP receptor-blocking agent, and oral anticoagulant treatment at inclusion and at the early follow-up visit. Model 3 included model 1 and model 2. All hazard ratios reflect the effect of a one standard deviation increase.

Thrombin peak and ETP were not associated with the composite endpoint either at inclusion or at early follow-up (Supplement, available online). Higher thrombin peak at early follow-up was associated with increased risk of all-cause mortality (Figure 4) and reduced risk of all-cause stroke (HR 0.50 [95% CI 0.30–0.81], *P* = 0.005). Similar results were found for ETP at early follow-up (Figure 5). There were no associations between thrombin peak or ETP and risks of MI or CHF (Figures 4 and 5).

Also after adjustment for baseline characteristics, thrombin peak and ETP were both associated with reduced risk of all-cause stroke. However, adjusting for antithrombotic treatment attenuated the effect of thrombin peak and ETP for all-cause stroke prediction (Figures 4 and 5).

Changes in thrombin peak and ETP from inclusion to early follow-up were not associated with either the risk of the composite endpoint or the risk of the individual cardiovascular endpoints.

The markers for thrombin generation potential were not related to the risk of clinically relevant bleeding (Figures 4 and 5).

## Discussion

In this study of unselected MI patients treated according to contemporary guidelines, new cardiovascular and bleeding events were associated with D-dimer concentrations measured 2–3 weeks after discharge, but not with D-dimer concentrations measured early after the index MI. The thrombin generation potential was not associated with the composite of cardiovascular or bleeding events but was inversely associated with the risk of all-cause stroke.

Higher D-dimer concentrations in patients with acute coronary syndromes have previously been associated with increased risks of cardiovascular events during follow-up (2,11). When these previous studies were performed, however, the recommended acute treatment mainly consisted of antithrombotic therapy with low-molecular-weight heparins and aspirin. In the present study, where patients were included and first blood sample obtained within in median two days after an acute MI, we found no significant associations between initial D-dimer concentrations and future risk of cardiovascular events. At this very early stage after an acute MI, a patient’s coagulation activity might be influenced by the coronary plaque rupture, by the percutaneous coronary intervention, or by the initial treatment with parenteral anticoagulant drugs (12,13). Indeed, an early dynamic change of D-dimer concentrations influenced by the acute treatment and the initial cellular stress has been shown in STEMI patients (14). Therefore, evaluation of D-dimer concentrations very early after an MI might not be adequate for identifying patients with persistent high coagulation activity.

In contrast, D-dimer concentrations measured 2–3 weeks after discharge in the present study were elevated above the reference interval in the majority of patients, and D-dimer concentrations at this time-point were related to the future risk of new cardiovascular events, including all-cause death, new MI, and CHF. This association persisted after adjustment for antithrombotic treatment in relation to the index event. Almost all patients in the present study were treated with a dual antiplatelet regime, which has not been shown to influence D-dimer concentrations in patients with coronary artery disease, but very few were treated with oral anticoagulants (4,15).

Higher D-dimer concentrations were associated with hospitalization for CHF in the present study. CHF has previously been associated with increased platelet activity and increased risks of ischemic stroke and venous thromboembolism (16,17). Hospitalization for CHF after MI is common, and high D-dimer concentrations at admission have been associated with increased in-hospital mortality in patients with acute decompensated heart failure (18). Further studies exploring potential mechanisms behind the association of D-dimer concentrations and the risk of CHF found in this study are warranted.

Higher D-dimer concentrations were also associated with increased risk of clinically relevant bleeding during follow-up, in accordance with previous results in STEMI patients (19). D-dimer reflects fibrin turnover, and formation of fibrin is important to prevent bleeding (20). Enhanced fibrin turnover resulting in increased D-dimer concentrations might therefore be disadvantageous (21). Higher D-dimer concentrations were further associated with older age, complex comorbidities, and with inflammatory state, and might thus reflect patient frailty (22).

D-dimer concentration measurements at follow-up early after MI may be an additional tool for identification of patients at higher risk of new atherothrombotic and/or bleeding events, but potentially also for selection of additional treatment. Vitamin K antagonists as well as new oral anticoagulant (NOAC) drugs modulate D-dimer concentrations during acute thromboembolic events (23). Furthermore, the addition of oral anticoagulant treatment has been reported to reduce the risk of recurrent events after MI to a higher degree than antiplatelet drugs alone (5,24). Patients with higher D-dimer concentrations might thus benefit from the addition of oral anticoagulant treatment. Further studies evaluating the effects of the addition of oral anticoagulant treatment to guideline-based treatment in patients with a recent MI and high D-Dimer concentrations are needed to evaluate if the possible benefit on thromboembolic events outweighs harm due to bleeding events.

The thrombin generation potential in the present study was evaluated by thrombin peak and ETP, and these markers are related to plasma levels of prothrombin fragment 1 + 2 (25,26). Thrombin peak and ETP at early follow-up were both influenced by OAC as well as by ACEi/ARB treatment, indicating the interplay between vascular and coagulation systems in thrombin generation [reviewed by Kalz et al. (27)].

Similar to the D-dimer concentration results, higher thrombin generation potential evaluated at early follow-up was associated with increased risk of all-cause death in this study. Thrombin generation potential, measured as ETP, has been associated with increased atherosclerotic burden, which may contribute to these results (28).

In contrast, both thrombin peak and ETP were inversely associated with the risk of future all-cause stroke, even after multivariable adjustment for baseline characteristics. A similar inverse association between thrombin generation potential and all-cause stroke was recently described in elderly patients with vascular disease (29). Low thrombin generation has previously been associated with higher risk of new cardiovascular events after STEMI and in patients with peripheral arterial disease (26,30). The biological mechanisms explaining these findings are not fully known. Tissue factor pathway inhibitor, formed upon plaque rupture or vessel injury, determines the thrombin generation potential *ex vivo* in MI patients (26). This might explain the association of low thrombin generation potential and increased risk of all-cause stroke in the present study. The time-point for evaluating the thrombin generation potential after an acute event could also be important due to the endogenous regulation of coagulation activity and the consumption of involved proteins. Further studies are needed to explore these biological mechanisms, and to increase the knowledge and interpret the results when measuring the thrombin potential *ex vivo* without the normal composition of coagulation and inflammation regulators found in whole blood.

This study has some limitations. First, albeit this unselected study cohort was similar in age and sex distributions to the general MI population in Sweden, there were some notable differences: the present population had an unusually large STEMI proportion (10); the mortality rate during the first two years of the present study was lower than expected (31,32); and the proportions of patients undergoing of revascularization and receiving dual antiplatelet therapy were higher than average (1). Second, the frequency of cardiovascular events was lower than expected, thus limiting our ability to identify significant associations between coagulation activity markers and the individual endpoints. Third, the Calibrated Automated Thrombogram method for thrombin generation potential analysis is sensitive to heparins and oral anticoagulant treatment even after cessation, which inhibited thrombin formation (25,33,34). In addition, the protocol used in the present study did not include addition of tissue factor to induce thrombin generation (9). These factors might have contributed to no formation of thrombin, leading to flat thrombin curves in some samples.

In conclusion, patients with high D-dimer concentrations 2–3 weeks after myocardial infarction had increased risk of new cardiovascular and/or bleeding events. High thrombin generation potential at the same time-point was associated with reduced risk of all-cause stroke. Further studies on this patient group are warranted to evaluate whether modifying high D-dimer concentrations with antithrombotic treatment could reduce the frequency of new cardiovascular events without increasing bleeding events. The biological mechanisms regulating the thrombin generation potential *ex vivo* and the inverse association to the risk of all-cause stroke have to be further investigated.

## Supplementary Material

Supplemental data
